# Coronary microvascular dysfunction in HFpEF: pathophysiologic mechanisms, diagnosis, treatments, and the amplifying potential of sepsis

**DOI:** 10.3389/fmed.2026.1812235

**Published:** 2026-09-09

**Authors:** Sharnvir Chattha, Kush Kapadia, Daniel Lynch, Yi McWhorter

**Affiliations:** 1 Department of Internal Medicine, Sunrise Health GME Consortium, HCA Healthcare, Las Vegas, NV, United States; 2 ATSU-Kirksville College of Osteopathic Medicine, Kirksville, MO, United States; 3 Department of Intensive Critical Care, Sunrise Health GME Consortium, HCA Healthcare, Las Vegas, NV, United States

**Keywords:** CMD, coronary microvascular dysfunction, HFPEF, phenotype, precision medicine, sepsis, septic shock

## Abstract

Heart failure with preserved ejection fraction (HFpEF) is a heterogeneous syndrome in which coronary microvascular dysfunction (CMD) emerges as a central, prevalent pathobiologic substrate across multiple phenotypic clusters. CMD in HFpEF arises from intertwined mechanisms including systemic inflammation, impaired NO-cGMP-PKG signaling, microvascular rarefaction and fibrosis, mitochondrial dysfunction, and ventriculoarterial decoupling that together promote subendocardial ischemia, diastolic stiffness, reduced reserve, and adverse outcomes. Advances in diagnostics, both invasive and non-invasive, as well as individualized medical and surgical intervention, create avenues of opportunity to prevent CMD-mediated HFpEF at a precise level. Sepsis and septic shock can superimpose an acute second hit on this chronically injured microvasculature, precipitating acute decompensation and potentially accelerating long-term CMD-driven remodeling in vulnerable HFpEF patients. While mechanistically plausible, this association remains hypothesis-generating. Advanced hemodynamics and diagnostics with tailored vasopressor and inotropic management need to be better studied in complex states of septic shock in HFpEF patients to understand the influence of CMD in such states.

## 1 Introduction

Precision medicine aims at tailored treatment based on individualized pathology rather than algorithmic protocol. Unlike more homogeneous syndromes such as heart failure with reduced ejection fraction (HFrEF), this goal becomes particularly important in heterogeneous syndromes such as heart failure with preserved ejection fraction (HFpEF) (1, 2). Probability-based proteomic analyses grounded in machine learning can delineate heterogeneous syndromes such as HFpEF into phenotypic clusters (1, 2). Pathophysiologic nuances and their contributions to one or multiple phenotypes can then be analyzed with the goal of creating nuanced treatment models (1, 2).

In several phenotypes of HFpEF, coronary microvascular dysfunction (CMD) has demonstrated epidemiological prevalence and multiple mechanistic contributions (3–7). Sepsis and septic shock can potentiate these pathophysiologic mechanisms and deliver a second hit, producing an acute-on-chronic progression of CMD in HFpEF populations, meaning an acute insult superimposed on established, chronic microvascular injury, which may carry transient or long-term sequelae (8–10).

After briefly summarizing the concept and burden of CMD, we review five pathophysiologic associations to HFpEF and how sepsis may aggravate these associations through a theoretical second hit. We then detail diagnostic approaches for CMD and HFpEF while highlighting newer technologies that allow for assessment of sepsis in CMD-HFpEF populations. Finally, we review current literature on medical and surgical intervention in HFpEF as it relates to CMD within the scope of precision medicine, and we consider vasopressor management in this population during septic shock.

## 2 Part 1: a review of coronary microvascular dysfunction and HFpEF heterogeneity

**Figure 1:**
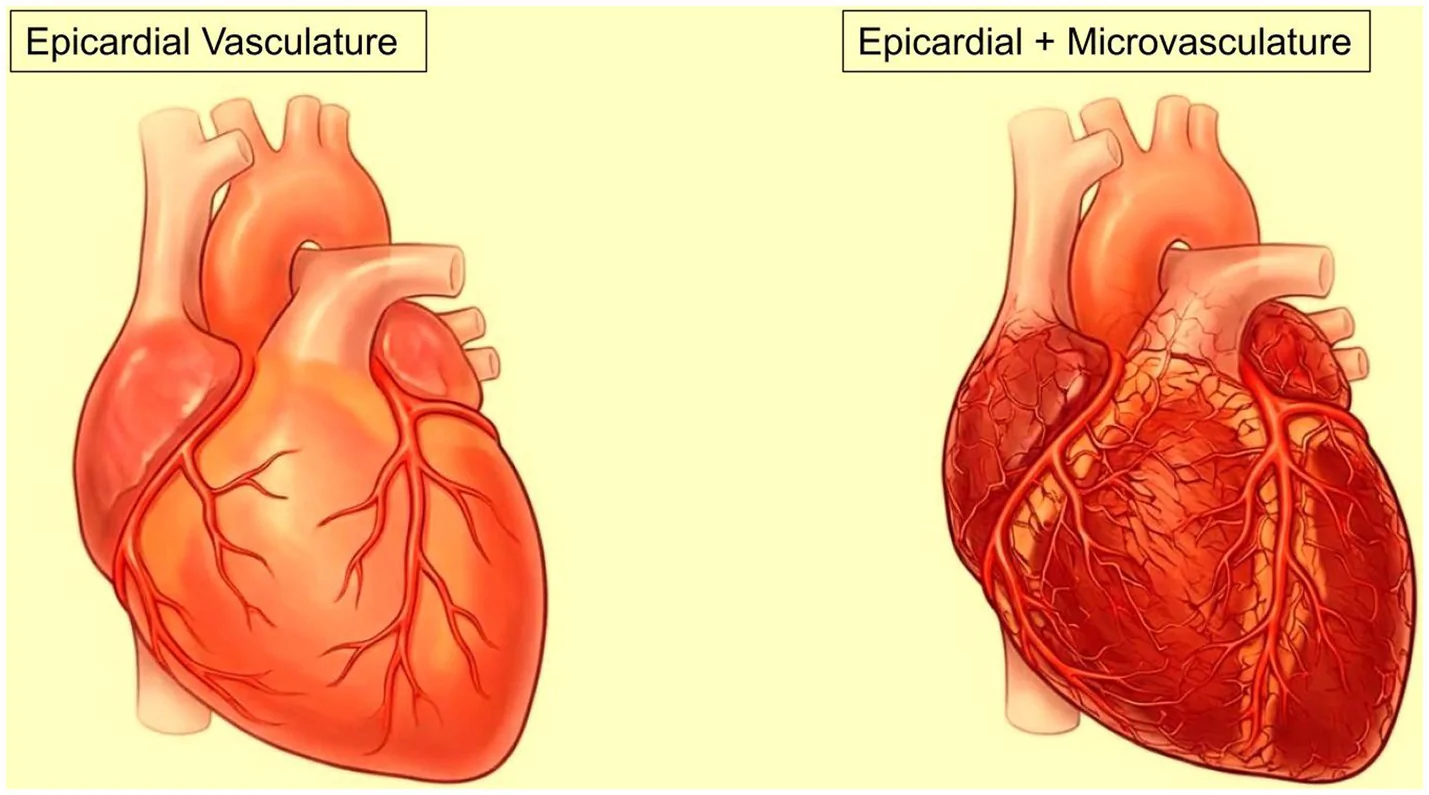
Epicardial versus microvascular contributions to coronary circulation. Schematic comparison of the coronary vasculature visualized by conventional angiography versus the full coronary tree. The left side shows the epicardial arteries alone, which are the vessels resolved on invasive coronary angiography and which account for only a small fraction of total coronary resistance. The right side superimposes the pre-arterioles, arterioles, and capillaries, which lie below the spatial resolution of angiography yet constitute the principal site of coronary flow regulation.

The complexity of cardiac anatomy and the epidemiological burden of chest pain has driven investigation beyond the visible epicardial components (approximately 0 to 10%) typically seen during angiography, toward the pre-arterioles (approximately 20%), arterioles (approximately 60%), and capillaries (approximately 10 to 15%) (11, 12). Dysfunction in this vasculature has been termed coronary microvascular dysfunction (CMD) [Figure 1]. Physiologically, the epicardial arteries are the primary conduits for conductance, but microcirculatory dysfunction limits the adaptability of the coronaries to augment blood flow in response to demand, given the heavy influence of the microcirculation on resistance (approximately 85%) (1–3, 12). Such a mismatch manifests as clinical or subclinical myocardial ischemia, informally termed non-obstructive coronary artery disease, and further grouped into the sub-categories of myocardial infarction with non-obstructive coronary arteries (MINOCA), ischemia with non-obstructive coronary arteries (INOCA), and angina with non-obstructive coronary arteries (ANOCA) (13).

CMD, in relation to non-obstructive coronary artery disease and primarily INOCA, is defined by a coronary flow reserve (CFR) below 2 and an index of microvascular resistance (IMR) above 25, or an endothelial-dependent CFR below 1.5, in the absence of obstructive coronary artery disease (13–17). Testing with adenosine and acetylcholine challenges during a functional angiogram, elaborated later, delineates CMD into structural and functional derivatives (15, 16). In the setting of increased myocardial oxygen demand, normal physiology dictates adenosine-mediated vasodilation in the microvasculature that is primarily endothelial-independent (4, 13–15). This pathway also leads to increased myocardial blood flow, stimulating acetylcholine release, which triggers endothelial-dependent nitric oxide (NO) release and further vasodilation (4, 14). Typically, acetylcholine-induced NO release from the endothelium predominates over the acetylcholine-induced vasospastic effects on smooth muscle, creating an overall vasodilatory effect (4, 13–17).

Structural CMD, also termed microvascular angina or endothelial-independent CMD, encompasses the former part of the definition: CFR below 2 and IMR above 25 during functional angiogram testing with adenosine (17). Proposed mechanisms include vascular remodeling and rarefaction, luminal obstruction, perivascular fibrosis and inflammation, and systemic inflammation from oxidative stress. Functional CMD, also termed microspastic angina or endothelial-dependent CMD, encompasses the latter component of the definition, with an endothelial-dependent CFR below 1.5, or less than 50% augmentation of myocardial blood flow, during functional angiogram testing with acetylcholine (15–17). In functional CMD, IMR is typically normal and patients have a paradoxically higher resting myocardial blood flow, either as a compensatory response to increased myocardial demand at rest or from disordered regulatory mechanics involving neuronal nitric oxide synthase (4, 5, 13–15). Because of these functional alterations, patients have a maximized vasodilatory state at rest, leading to minimal or attenuated vasodilatory capability, or even a shift toward transient vasoconstriction with acetylcholine under physiologic stress. Mechanisms vary and include endothelial dysfunction, vasomotor imbalance, autonomic dysregulation, impaired NO signaling, and Rho-kinase activity (3–5, 15, 17).

The vascular stiffening phenogroup is of particular relevance to CMD. Increased central arterial and pulsatile load raises arterial elastance and augments systolic wall stress which, together with elevated left ventricular diastolic pressures, reduces the gradient driving subendocardial perfusion and lowers coronary flow reserve. This converges on the ventriculoarterial decoupling mechanism detailed in the fifth link of Part 2. Consistent with a predominantly structural substrate, hospitalized HFpEF cohorts show that structural, endothelium-independent CMD, defined by reduced CFR with elevated IMR, is more prevalent (approximately 66%) than the endothelium-dependent functional form (approximately 24%). The two commonly coexist, and their relative contribution likely shifts across disease stages as microvascular remodeling, rarefaction, and perivascular fibrosis accumulate (15, 18).

### 2.1 Heterogeneity of HFpEF and its associated relation to CMD

Unlike HFrEF, which is commonly characterized as a homogeneous syndrome with generalized treatment recommendations, HFpEF has been characterized as a heterogeneous syndrome with distinct clinical and pathophysiologic phenotypes (1, 2). Through statistical learning algorithms, hierarchical cluster analysis, and penalized model-based clustering, patients with HFpEF can be phenotypically clustered into differing patterns of pathophysiology based on probability derivations (1, 2). By establishing such a framework, varied mechanisms such as CMD or chronotropic incompetence can be studied independently for their effect on patient symptoms and outcomes, contributing to the goal of targeted therapeutic strategies in precision medicine (1, 2).

Multiple, overlapping phenotypic frameworks of HFpEF have been proposed, and several involve or overlap with CMD. Shah et al. derived three probability-based clusters through phenomapping: natriuretic peptide-deficient (obesity-predominant), cardiometabolic, and right ventricular failure/cardio-abdominal-renal (2). Subsequent deep phenotyping work has since described additional, non-mutually exclusive clinical trait phenogroups, including pulmonary vascular disease, left atrial myopathy, and vascular stiffening (6, 7). Among 643 patients undergoing invasive exercise testing, 94% met criteria for at least one phenogroup and roughly two-thirds met criteria for several, with greater phenogroup multiplicity associated with worse exercise hemodynamics and a higher risk of heart failure hospitalization or death (7). CMD contributes to several of these phenogroups but has been characterized most directly in the cardiometabolic and vascular phenotypes. Therefore, we focus this review on three representative clusters, the role of CMD within them, and their potential exacerbation by sepsis. This is a deliberate scope limitation rather than an assertion that only three phenotypes exist.

CMD was noted to be a forerunner among the mechanisms in all three phenomapping phenotypes, but most particularly the cardiometabolic subtype (1, 2, 19, 20). Numerous studies, most notably the PROMIS-HFpEF trial, and a subsequent meta-analysis revealed a clear associative link between HFpEF and CMD in approximately 70 to 75% of patients (15, 20). CMD in HFpEF is consistently associated with greater disease severity and significantly higher risks of heart failure hospitalization, major adverse cardiovascular events, readmissions, and mortality (15).

### 2.2 Sex differences in HFpEF and CMD

Sex is an important and under-emphasized modifier of the mechanisms described here. HFpEF is more prevalent in women, who more often show concentric left ventricular remodeling, pronounced diastolic dysfunction, and greater central arterial stiffening than men (21, 116). CMD also shows a female predominance, and ischemic symptoms in women are disproportionately attributable to microvascular rather than obstructive disease (21, 22, 116, 117). Sex hormones plausibly link these observations. Estrogen favorably modulates coronary microvascular tone and attenuates sympathetic activity, and its loss after menopause is associated with increased susceptibility to CMD and endothelial dysfunction (22, 116, 117). Estrogen deficiency alone does not fully explain the sex disparity in HFpEF, which is also shaped by comorbidity patterns such as obesity and diabetes and by the resulting inflammatory and metabolic milieu (21, 116). A clinical corollary is the sex-by-treatment interaction seen with sacubitril/valsartan in PARAGON-HF, in which women appeared to derive greater benefit (23). With respect to sepsis, sex differences in incidence and outcome are recognized in the general critical care literature, but sex-specific data at the intersection of HFpEF, CMD, and sepsis are essentially absent. This is a clear and actionable gap for precision-medicine research.

It should be noted that the original phenomapping analysis reported the demographic composition of each cluster but did not establish a validated sex-specific mechanistic mapping of the three phenotypes (2). The obesity and cardiometabolic phenotypes are enriched for women, but a formal phenotype-by-sex correspondence is not yet supported by available data.

## 3 Part 2: the pathophysiologic links between CMD, HFpEF, and sepsis

### 3.1 Empirical basis and current evidence

The direct evidence base at the intersection of HFpEF, CMD, and sepsis is limited, and we state this once here rather than repeatedly throughout. What exists is as follows. Sublingual microcirculation studies in sepsis demonstrate reduced capillary density, increased numbers of non-perfused capillaries, and marked flow heterogeneity that is more severe in non-survivors (10). A small cardiac magnetic resonance imaging cohort of 21 adults documented coronary microvascular dysfunction during septic shock that improved or resolved at follow-up, indicating that sepsis-associated CMD is frequently transient (24). Direct measurement of myocardial blood flow in sepsis has confirmed altered perfusion despite preserved ejection fraction (25). Pharmacologic data provide indirect mechanistic support, as methylene blue, a non-specific nitric oxide synthase inhibitor, reduces inotropic requirements and improves mean arterial pressure and stroke volume in septic patients, although without demonstrated effect on outcomes (26). No study to date has directly measured CMD indices in a HFpEF-specific septic cohort. The mechanistic links that follow are therefore presented as hypothesis-generating rather than as established associations, and each is anchored to the evidence cited above.

The presence of cardiovascular dysfunction in sepsis is associated with a substantially increased mortality rate compared with septic patients without cardiovascular impairment (27–31). In sepsis, patients with preexisting HFpEF appears more vulnerable than non-heart-failure patients early on, with higher emergency department mortality, earlier intubation, and often more conservative fluid resuscitation, although their adjusted in-hospital mortality is not clearly higher (27–31). Overall, the data suggest that while both HFpEF and HFrEF complicate sepsis care, HFrEF confers a distinctly higher short-term risk (27–31). Current practice is guided more by physiologic judgment than by phenotype-specific evaluation, underscoring the need for dynamic assessment and tailored fluid and vasopressor strategies in a heterogeneous syndrome such as HFpEF.

Current data cannot tell us why patients with preexisting HFpEF are more vulnerable early in sepsis. One possibility is a direct effect of the HFpEF microvascular substrate. Another is that the association is largely mediated by the shared cardiometabolic comorbidity burden, since obesity, diabetes, hypertension, and chronic kidney disease both characterize much of the HFpEF population and independently worsen sepsis outcomes. The available studies are observational and generally unadjusted for HFpEF phenotype, so the two cannot be separated. The cardiometabolic phenotype in particular may confound the association. This ambiguity reinforces the need for phenotype-aware, prospective evaluation rather than treating HFpEF as a single risk marker in sepsis.

**Figure 2:**
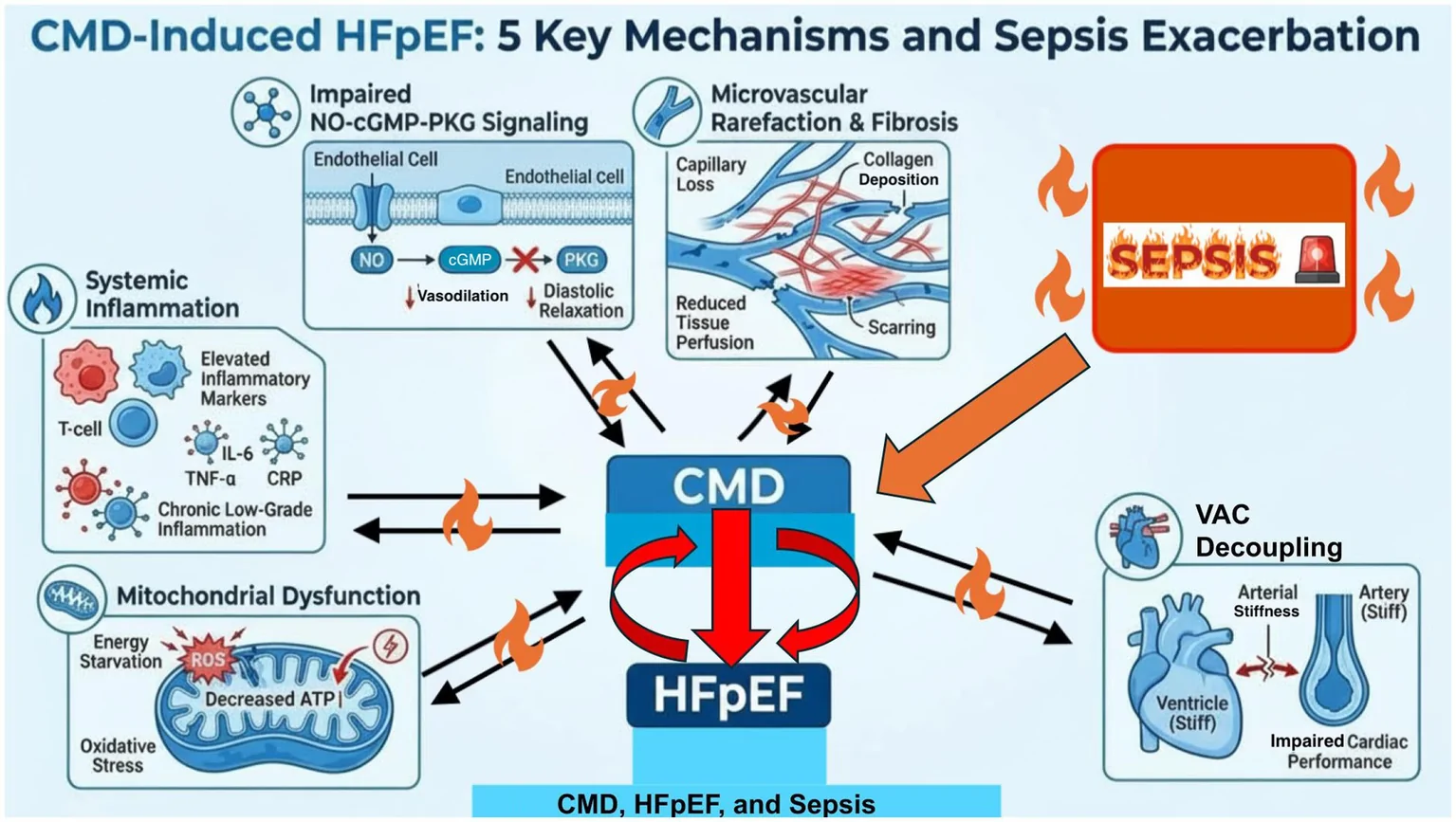
Coronary microvascular dysfunction in HFpEF: five key mechanisms and sepsis exacerbation. Conceptual overview of the mechanisms linking coronary microvascular dysfunction (CMD) and HFpEF, with the proposed amplifying role of sepsis. CMD and HFpEF occupy the centre in a bidirectional relationship, each reinforcing the other. Five mechanistic domains converge on this axis: systemic inflammation (elevated inflammatory markers, including interleukin-6, tumour necrosis factor-alpha, and C-reactive protein, with chronic low-grade inflammation); impaired nitric oxide–cyclic GMP–protein kinase G (NO–cGMP–PKG) signalling, with reduced vasodilation and impaired diastolic relaxation; microvascular rarefaction and fibrosis (capillary loss, collagen deposition, scarring, and reduced tissue perfusion); mitochondrial dysfunction (energy starvation, reduced ATP, oxidative stress, and reactive oxygen species); and ventriculoarterial decoupling (increased arterial and ventricular stiffness with impaired cardiac performance). Sepsis is depicted as a superimposed insult acting upon these mechanisms, representing the hypothesized “second hit” upon an already-injured microvasculature. CRP, C-reactive protein; IL-6, interleukin-6; TNF-α, tumour necrosis factor-alpha.

We propose sepsis and septic shock as a potential second hit that produces acute mechanistic changes in the microvasculature and may worsen the chronic first-hit dynamics of CMD in HFpEF. Coronavirus disease has been linked to progressive acute-on-chronic CMD changes, suggesting that the microbiologic cause of sepsis may itself introduce further variance (32). Although not exhaustive, we outline five interconnected pathobiologic mechanisms of association, encompassing both structural and functional abnormalities of CMD, that illustrate how CMD in HFpEF might be potentiated by sepsis [Figure 2].

#### 3.1.1 First link: systemic inflammation from comorbidities

A hypothesis stemming from the ALLHAT trial highlighted the first known link between comorbidities such as body mass index and compromised heart function (33). This hypothesis evolved into the Paulus paradigm, which details how metabolic syndrome, including diabetes, obesity, and hypertension, triggers a systemic inflammatory state in multiple organs, identified through markers such as interleukin-1 receptor-like 1, C-reactive protein, growth differentiation factor 15, tumor necrosis factor alpha (TNF-alpha), soluble ST2, and pentraxin-3, including the heart, leading to deranged endothelium-cardiomyocyte signaling (34, 35). Compounding derangements result in myocardial fibrosis, hypertrophy, and cardiomyocyte stiffening, all tenets of the cardiometabolic phenotype of HFpEF (3–5, 11, 34, 35). The PROMIS-HFpEF trial identified these derangements as a pathophysiologic sequela of CMD, confirmed through compromised CFR, which was evident in 75% of individuals with HFpEF (20). Proteomic analysis identified a network ranking of inflammation in HFpEF centered around TNF receptor 1 (2, 35, 36). Elevated filling pressures, demonstrated through effects on mitral E velocity and tricuspid regurgitation velocity in mediation analysis, support a mechanism in which TNF-alpha leads to downstream interstitial fibrosis and left ventricular stiffening through T-cell regulated biologic mechanisms (36).

Sepsis is a canonical pro-inflammatory state. A TNF-alpha and interleukin-1 beta driven cytokine response increases leukocyte adhesion, promotes microvascular injury, and may compound the TNF-alpha and TNF receptor 1 centered inflammatory signature described in HFpEF (8, 9, 36). Direct evidence linking sepsis-associated inflammation to CMD specifically in HFpEF patients is lacking. Therefore, the relationship is mechanistic and hypothesis-generating, inferred from concordant observations in each condition separately rather than from studies of the combination.

#### 3.1.2 Second link: reduced NO bioavailability and impaired NO-cGMP-PKG signaling

While interconnected with and largely driven by systemic inflammation, NO and the NO-cGMP-PKG pathway carry a more functionally mediated compromise of microvascular function that is endothelial-dependent.

Preclinical models of cardiometabolic HFpEF (ZSF1 rats) and, separately, human endomyocardial biopsy studies have each demonstrated downregulation of the NO-cGMP-PKG pathway in HFpEF (26).

Pathways of downregulation appear multifactorial and are postulated to involve oxidative stress and protein modification through nitrosative stress (3, 26). Biochemically, the pathway is straightforward. NO, synthesized by endothelial nitric oxide synthase to maintain hyperemic myocardial blood flow, leads to vasodilation, initiating the pathway. Endothelial-dependent CMD, identified by an endothelial-dependent CFR below 1.5, has a clear mechanistic link to HFpEF through disruption of physiologic vasodilation, such as during exercise, needed to maintain myocardial blood flow. While predominantly a functional compromise, downregulation of this pathway also has structural effects through pro-hypertrophic and pro-fibrotic mechanisms such as N2B titin hypophosphorylation, which can lead to structural cardiac remodeling in HFpEF (26, 35).

NO is not the sole mediator of endothelium-dependent relaxation in the coronary microcirculation. Endothelium-derived hyperpolarizing factors (EDHFs), which include cytochrome P450-derived epoxyeicosatrienoic acids, hydrogen peroxide, and electrotonic conduction through myoendothelial gap junctions acting via calcium-activated potassium channels, contribute proportionally more to endothelium-dependent vasodilation as vessel caliber decreases. EDHF can also serve as a compensatory backup when NO bioavailability falls, and there is reciprocal cross-talk between the two pathways. Experimental work links impaired endothelium-dependent hyperpolarization to both coronary microvascular dysfunction and diastolic dysfunction, and inflammatory signaling can alter EDHF-mediated responses (37, 113). What remains poorly defined is the quantitative contribution of EDHF within the human HFpEF microcirculation, and how chronic inflammation, or superimposed sepsis, shifts the balance between NO-dependent and EDHF-dependent vasodilation. This is an important and largely unexplored dimension of microvascular endothelial dysfunction in HFpEF and warrants dedicated study.

Sepsis, through oxidative and nitrosative stress involving inducible nitric oxide synthase induction and peroxynitrite formation, may deepen existing deficiencies of this axis through NO scavenging and endothelial nitric oxide synthase uncoupling (3, 8, 9, 26). Indirect support comes from the observation that methylene blue, a non-specific nitric oxide synthase inhibitor, decreases inotropic requirements and improves mean arterial pressure and stroke volume in septic patients, without demonstrated effect on outcomes (26). This link remains mechanistic and has not been tested in HFpEF-specific septic cohorts.

#### 3.1.3 Third link: microvascular rarefaction, myocardial fibrosis, and reduced hyperemic flow

Microvascular rarefaction, primarily within the capillary bed of the coronaries, limits perfusion reserve and promotes low-grade microvascular ischemia in HFpEF, leading to reduced CFR and higher IMR in line with the structural form of CMD (3, 35, 38). Perivascular fibrosis contributes to cardiovascular remodeling, resulting in the impaired relaxation seen in HFpEF (3, 35, 38). More specifically, chronic CMD and inflammation activate fibroblasts and transforming growth factor beta signaling, leading to fibrosis and rarefaction (35). Autopsy studies, confirmed by an invasive coronary study, highlight vascular rarefaction as a potential late-stage manifestation of HFpEF leading to increased IMR and compromised CFR that may not be present in early or acute HFpEF phenotypes (38).

Sepsis has been shown to cause profound microvascular flow heterogeneity with diminished microvascular reserve (10, 24). Endothelin-1, a potent vasoconstrictor elevated in both sepsis and HFpEF, causes microvascular vasoconstriction, leading to transient shunting and rarefaction (39, 40). Septic microthrombi and interstitial edema producing microvascular compression can cause a similar effect. Microcirculation studies show this rarefaction-related CMD to be largely transient (10, 24). In a HFpEF patient with fixed rarefaction and diminished reserve, this acute insult could plausibly produce more severe ischemia, potentially mediated through profibrotic signaling. This remains a mechanistic hypothesis and has not been demonstrated directly.

#### 3.1.4 Fourth link: oxidative stress, mitochondrial dysfunction, and impaired bioenergetics

Using the phosphocreatine-to-ATP ratio, animal models have demonstrated a clear energy deficiency in HFpEF (41). Cardiomyocytes are metabolically flexible and able to use different substrates to generate ATP, but biochemical variation is commonly seen in substrate use across the ejection fraction spectrum (42). HFrEF typically shows a shift toward carbohydrates and ketones, while HFpEF preferentially prioritizes fatty acids (42). This shift has been linked to high-fat diets and obesity-based comorbidities in HFpEF. Fluorescent spectral analysis and mitophagy flux analysis illustrated an atypical downregulation of mitophagy in HFpEF that was not similarly seen in high-fat diet controls (42). Induced pluripotent stem cell-derived cardiomyocyte analysis illustrated a conundrum in HFpEF: Fatty acids were the preferred fuel source and accumulated in HFpEF myocardium, but fatty acid oxidation did not increase sufficiently to match supply, leading to decreased mitophagy. 37 (42), Impaired myocardial energetics in HFpEF therefore stems in part from inadequate fatty acid oxidation, leading to mitochondrial dysfunction.

Mitochondrial dysfunction in turn contributes to the development of CMD through oxidative stress from proton leakage, ATP supply and demand mismatch, and mechano-energetic decoupling involving alterations in the mitochondrial calcium uniporter (3, 35, 43). Because of these alterations, myocardial blood flow cannot match myocardial demand, resulting in microvascular ischemia (3, 35, 43). This sustains a bidirectional cycle in which mitochondrial dysfunction driven by comorbidities and obesity in HFpEF contributes to the development of CMD, which then exacerbates diastolic dysfunction by impairing coronary flow reserve (3, 35, 43).

Sepsis triggers multiple biochemical routes toward mitochondrial injury in the heart and vessels, which can further damage microvascular endothelium and myocardial energetics (8, 9). Proposed mechanisms include compromised oxidative phosphorylation from reactive oxygen species, altered calcium transport leading to impaired ATP production, enhanced mitochondrial fission, and impaired mitophagy (8–10, 35, 42). Because HFpEF microvascular disease is already sensitive to mitochondrial dysfunction, sepsis could plausibly deepen energetic failure, although this has not been directly demonstrated in HFpEF cohorts.

#### 3.1.5 Fifth link: ventriculoarterial decoupling

Ventriculoarterial coupling describes cardiovascular function using arterial elastance (Ea), the arterial load the heart pumps against, derived from the three-element Windkessel model, and ventricular end-systolic elastance (Ees), a measure of ventricular contractility (44). The ratio of Ea to Ees, derived from pressure–volume loops, illustrates optimized function at approximately 1, where cardiovascular function is metabolically optimized by minimizing oxygen consumption and enhancing ventricular efficiency (44, 45). Like mitochondrial bioenergetics, ventriculoarterial coupling has a bidirectional relationship with CMD and HFpEF (11, 15, 44). Increased arterial stiffness and heightened pulsatile afterload in HFpEF result in a high Ea, leading to decoupling (11, 15). This produces excessive systolic wall stress and elevates myocardial oxygen demand while reducing coronary perfusion because of elevated left ventricular diastolic pressures (11, 15). Microvascular flow is thereby compromised, resulting in both structural deficiencies linked to CMD, including oxidative stress, inflammation, and microvascular rarefaction, and functional deficiencies including endothelial dysfunction and decreased NO bioavailability (11, 15). In turn, CMD fosters subclinical ischemia, myocardial fibrosis, and impaired relaxation, amplifying diastolic stiffness and reinforcing the HFpEF phenotype (11, 15).

Sepsis and septic shock create substantial variability in Ea and Ees. Myocardial depression drives both diastolic impairment and contractile suppression, while pathologic vasodilation combined with vasopressor administration drives arterial elastance (11, 15). Intensive care unit studies illustrate a tendency toward decreased Ees, mediated by septic cardiomyopathy, and increased Ea, driven by vasopressor administration, with ratios typically above 1.2 (44, 45). Specifics regarding HFpEF patients were not defined in these studies. Sepsis would plausibly result in more severe ventriculoarterial decoupling in HFpEF patients, primarily through arterial elastance, although the interplay between vasopressor pharmacology and individual patient physiology would likely produce varied presentations. This underscores the need for comprehensive hemodynamic assessment in this population.

## 4 Part 3: diagnostic evaluation of HFpEF and CMD in outpatient and acute settings

### 4.1 General HFpEF and CMD diagnostic tools and evaluation

**Figure 3:**
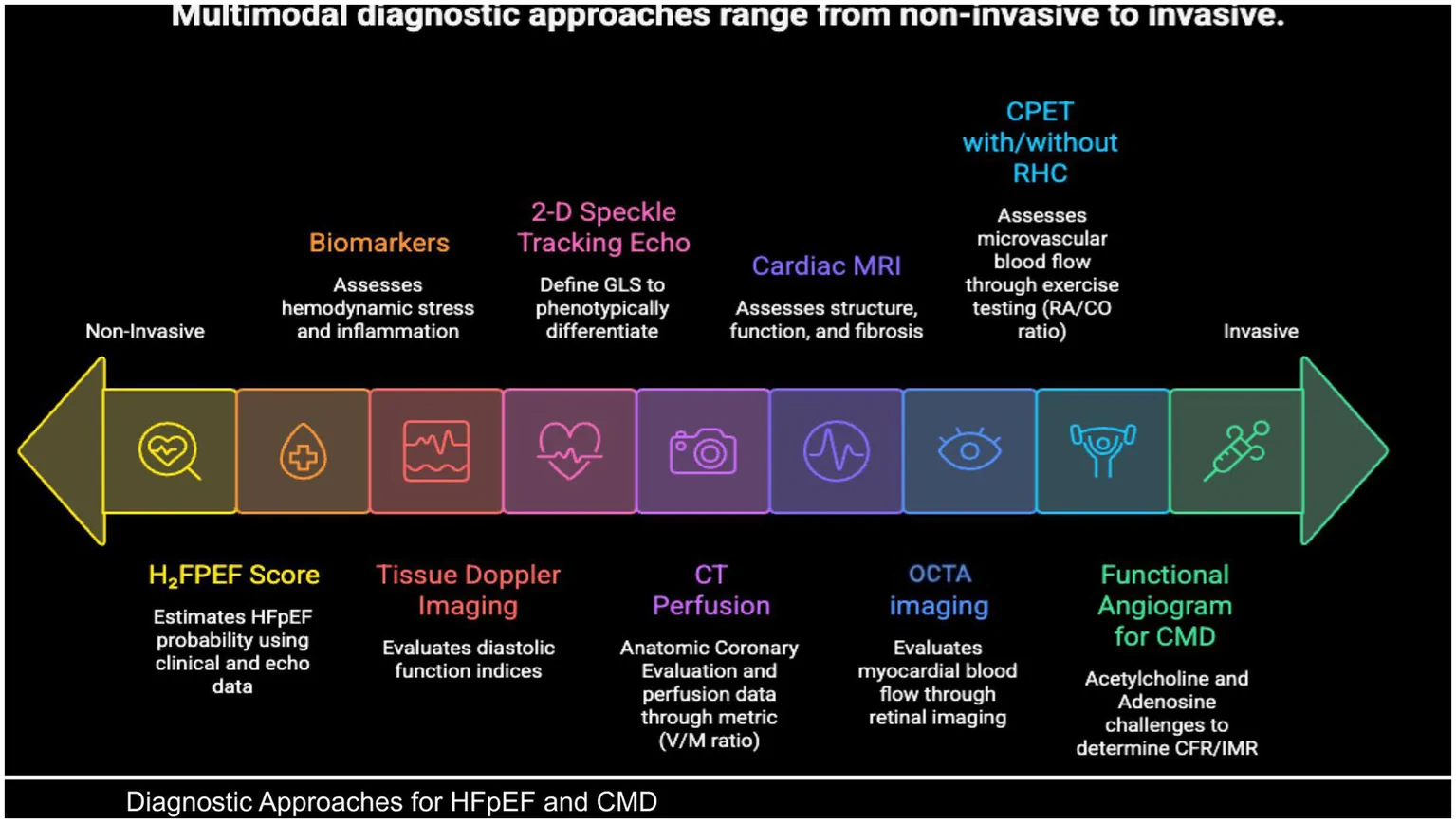
Multimodal diagnostic approaches to HFpEF and CMD, ranging from non-invasive to invasive. Spectrum of diagnostic modalities used to evaluate HFpEF and coronary microvascular dysfunction, arranged along a continuum from non-invasive (left) to invasive (right). Modalities shown are the H₂FPEF score, which estimates HFpEF probability from clinical and echocardiographic data; biomarkers, which assess haemodynamic stress and inflammation; tissue Doppler imaging, which evaluates diastolic function indices; two-dimensional speckle-tracking echocardiography, which defines global longitudinal strain to phenotypically differentiate; computed tomography perfusion, which provides anatomic coronary evaluation and perfusion data via the volume-to-mass ratio; cardiac magnetic resonance imaging, which assesses structure, function, and fibrosis; OCTA imaging, which evaluates myocardial blood flow through retinal microvascular imaging; cardiopulmonary exercise testing with or without right heart catheterization, which assesses microvascular blood flow through exercise testing; and functional angiography, which uses acetylcholine and adenosine challenge to determine coronary flow reserve and the index of microvascular resistance. CFR, coronary flow reserve; CMD, coronary microvascular dysfunction; GLS, global longitudinal strain; IMR, index of microvascular resistance; OCTA, optical coherence tomography angiography; RHC, right heart catheterization.

HFpEF requires individualized assessment and management. Various tools, including clinical assessment, biomarkers, echocardiography with tissue Doppler imaging, coronary and functional angiography, cardiac magnetic resonance imaging, and invasive or non-invasive hemodynamic assessment in various settings including shock, can help optimize diagnosis and guide treatment decisions [Figure 3] (2, 46–53). With the field moving toward precision medicine, categorizing HFpEF patients into phenotypic clusters using multidimensional diagnostic analysis has become increasingly standard.

Initial evaluation can be made with pre-test probability scores such as the H2FPEF score, based on pulmonary capillary wedge pressure, with a value above 15 mmHg considered diagnostic, and E/e’ ratios, with integrated clinical influences such as obesity and age (46). The passive leg raise test is also diagnostic if right heart catheterization shows a pulmonary capillary wedge pressure above 19 mmHg (47). Nuances in patient physiology, such as increased intrathoracic pressure in obese patients, led to modified slope curve analysis of right atrial pressure over cardiac output, with values greater than 1.3 considered abnormal (48). Similar slope analyses have also been validated, such as mean pulmonary arterial pressure over cardiac output above 3 (48). Phenotypic analysis extending beyond ejection fraction has resulted in evaluation of longitudinal strain through tissue Doppler imaging and two-dimensional speckle tracking echocardiography (2, 49–51). Assessment of s’, a’, and e’ velocities, as well as isovolumic and relaxation times, gives insight into myocardial compromise, distinguishing preserved from reduced longitudinal strain (40, 49–51). Speckle tracking is more specific in its ability to define global longitudinal strain, with normal values typically above 18% and abnormal characterized as below 16% (51). Echocardiography and speckle tracking provide primarily anatomical evaluation, while cardiopulmonary exercise testing, with or without right heart catheterization, provides more detailed functional evaluation during exercise through metrics such as oxygen consumption in relation to cardiac output (52). Through these functional analyses, mitochondrial and circulatory dysfunction can be more specifically characterized (52). Cardiac magnetic resonance imaging and genetic testing can serve a role in patients requiring more comprehensive assessment (53).

Given the significance of CMD and its associated morbidity, with more than 47% of affected patients retiring early, 38% applying for disability, and 20% reducing working hours, its contributory role in HFpEF should be routinely assessed (54). Traditional evaluation requires a functional angiogram using adenosine and acetylcholine with invasive guidewire-based measurement of CFR and IMR through thermodilution or Doppler methods (55, 56). Measurement of both is required, as CFR is non-specific and can be influenced by hemodynamics, while IMR can more strongly differentiate microvascular compromise through fixed assessments of pressure in thermodilution analysis (55, 56). Various interventional protocols implement an adenosine challenge first, followed by an acetylcholine challenge uptitrated to induce both epicardial and microvascular spasm (55, 56). Both challenges help delineate structural from functional CMD, which require individualized medical intervention as defined by the CORMICA trial (57).

While functional angiography is the gold standard for diagnosis, non-invasive assessment has become a large area of research. Contrast echocardiography with microbubble contrast and pulsed-wave Doppler assessment of the proximal left anterior descending artery is largely non-specific and operator-dependent but has advantages in cost, risk, and ease of use (53, 55, 57–60). Computed tomography perfusion provides anatomic coronary evaluation and perfusion data through metrics such as the coronary lumen volume to myocardial mass ratio but requires high radiation exposure and may overestimate myocardial blood flow (53, 55, 57–60). Cardiac magnetic resonance imaging uses dynamic first-pass vasodilation and subsequent rest imaging to gather cine images, T1 mapping, and late gadolinium enhancement sequences (53, 55, 57–60). It is most useful in assessing patients who have CMD with concomitant hypertrophic cardiomyopathy or amyloidosis (53, 55, 57–60). Positron emission tomography is the most validated non-invasive tool, similarly using dynamic first-pass vasodilation and rest imaging to measure hyperemic and resting myocardial blood flow and to derive metrics such as myocardial flow reserve and coronary vascular resistance (59, 60). Newer techniques attempt to evaluate microvasculature elsewhere in the body to assess for diagnostic corollaries with CMD. Nailfold capillaroscopy and optical coherence tomography angiography, the latter better studied, show potential (60, 61). Biomarkers rooted in neutrophil extracellular trap formation, such as citrullinated histone H3, also show potential, although these studies remain early (62).

### 4.2 Evaluation of CMD in HFpEF during sepsis

The complex interplay of CMD and HFpEF during sepsis and shock requires comprehensive ongoing assessment to optimize treatment in settings such as the intensive care unit. Assessment during these states is usually guided by invasive hemodynamics with parameters including venous oxygen saturation, cardiac index, pulmonary capillary wedge pressure, and central venous pressure, used to guide vasopressor and inotropic support (63). Newer technologies show potential for evaluating CMD with minimally invasive or entirely non-invasive approaches. Arterial line pressure waveform analysis allows for complex hemodynamic assessment of parameters such as dP/dt, dynamic arterial elastance, Ees, and Ea, which could shed insight into the role of septic shock on CMD in HFpEF (64, 65). These parameters have not, however, been evaluated in the context of CMD, and significant research is required. Orthogonal polarization spectral imaging shows promise as it uses polarized light and wavelength reflection to illuminate microvessels without contrast (10). One study demonstrated its utility in showing how sepsis reduces capillary density, resulting in rarefaction and microvascular compromise in the acute setting (10).

## 5 Part 4: medical and surgical intervention in CMD-HFpEF

### 5.1 Established pharmaceutical interventions in HFpEF as they relate to CMD

As noted in Part 1, HFpEF encompasses several overlapping phenogroups. For clarity of therapeutic mapping, we again organize the discussion around three representative clusters: natriuretic peptide-deficient (obesity-predominant), cardiometabolic (inflammatory), and right ventricular failure/cardiorenal. We recognize that the vascular stiffening and pulmonary vascular phenogroups likewise intersect with CMD and are relevant to several of the agents discussed below.

Phenotyping is a probability-based model, with patients typically exhibiting features of all three clusters (1, 2). For example, an individual patient’s physiologic analysis may probabilistically categorize them as 65% similar to one group, 30% to a second, and 5% to a third. CMD is mechanistically linked to all three, with greater associations established in the natriuretic peptide-deficient subtype through obesity and in the cardiometabolic subtype through inflammation (2).

**Figure 4:**
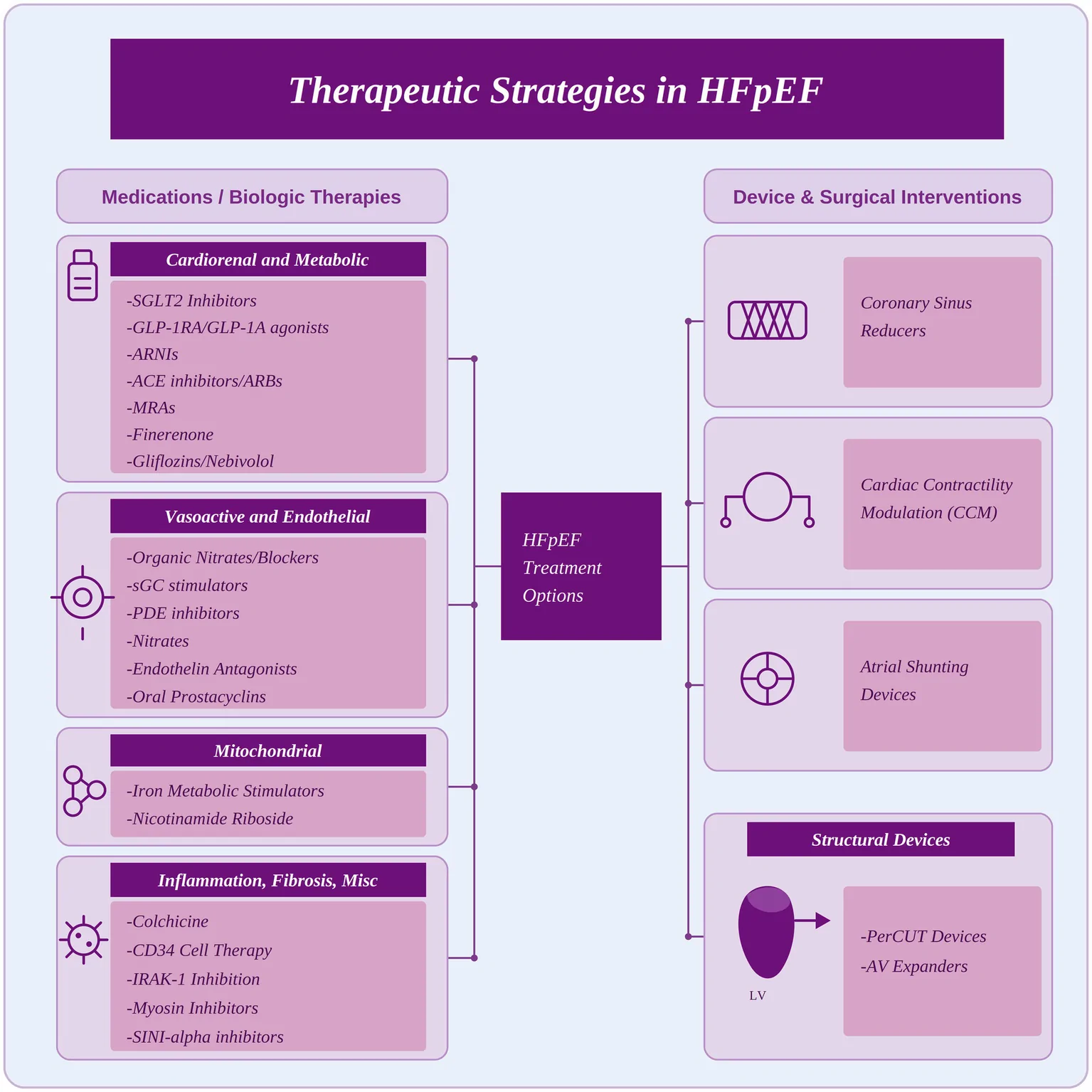
Therapeutic strategies in HFpEF relevant to coronary microvascular dysfunction. Overview of therapeutic options for HFpEF, organized around a central node of HFpEF treatment options and divided into medical or biologic therapies and device or surgical interventions. Medical and biologic therapies are grouped by mechanism: cardiorenal and metabolic agents (SGLT2 inhibitors; GLP-1 and GLP-1 receptor agonists and amylin agents; angiotensin receptor–neprilysin inhibitors; ACE inhibitors and ARBs; mineralocorticoid receptor antagonists; beta blockers, such as nebivolol); vasoactive and endothelial agents (calcium-channel blockers; soluble guanylate cyclase stimulators; phosphodiesterase inhibitors; nitrates; endothelin antagonists; oral prostacyclins); mitochondrial agents (the HU6 metabolic stimulator; nicotinamide riboside); and anti-inflammatory, antifibrotic, and other agents (colchicine; CD34+ cell therapy; urate transporter-1 inhibition; myosin inhibitors; and interleukin-6 inhibition). Device and surgical interventions comprise coronary sinus reducers, cardiac contractility modulation, atrial shunting devices, and structural devices, including pericardial resection devices and left ventricular expanders. ACE, angiotensin-converting enzyme; ARB, angiotensin II receptor blocker; GLP-1, glucagon-like peptide-1; SGLT2, sodium-glucose cotransporter-2.

Various agents [Figure 4] can be tailored based on HFpEF phenotype, but one class has proven utility across phenotypes. Sodium-glucose cotransporter-2 inhibitors, as seen in the EMPEROR-Preserved and DELIVER trials as well as various meta-analyses, consistently reduce cardiovascular death and heart failure hospitalization by up to 25% (66–68). These agents improve myocardial energy utilization and reduce cardiomyocyte stiffness, with large randomized trials demonstrating reductions in heart failure hospitalization and improvements in quality of life across the HFpEF spectrum (66–69). They also reduce inflammatory cytokines and fibrosis markers, with mechanistic work suggesting anti-inflammatory and antifibrotic effects relevant to HFpEF with CMD (66–69).

Metabolic and vascular-modifying therapies such as glucagon-like peptide-1 receptor agonists, glucagon receptor agonists, and amylin analogues further highlight phenotype-specific approaches, primarily toward the obesity-predominant subtype (2, 70–73). These agents improve endothelial function and reduce myocardial fibrosis, with contemporary trials such as STEP-HFpEF and SUMMIT demonstrating substantial improvements in symptoms, physical activity, and quality of life, along with decreases in body weight, C-reactive protein, and N-terminal pro B-type natriuretic peptide (70, 71). The SELECT trial reaffirms these findings in HFpEF populations by showing significant decreases in major adverse cardiovascular events and heart failure composites (72, 73). Although direct measures of coronary microvascular function remain limited, mechanistic links suggest improved myocardial reserve from decreased rarefaction and decreased systemic inflammation. Pericardial adipose tissue regression seen in the SUMMIT trial may also play a role in CMD through decreased arterial elastance and better ventriculoarterial coupling (71).

Angiotensin receptor neprilysin inhibitors currently carry a class 2a recommendation for HFpEF, with the PARAGON-HF trial narrowly missing its primary endpoint (23). The agent shows promise in specified subgroups, particularly women and patients with a lower ejection fraction within the HFpEF spectrum (23). The specific potential in CMD is unclear and likely indirect. By enhancing natriuretic peptide and cGMP signaling and reducing wall stress, these agents may improve endothelial function and microvascular hemodynamics, but trials have not directly demonstrated improved CFR or CMD endpoints (15, 26). Renin–angiotensin–aldosterone system inhibition through angiotensin-converting enzyme inhibitors or angiotensin II receptor blockers improves CFR in INOCA patients with CMD, but corresponding data in HFpEF patients with CMD are lacking (5, 74).

Mineralocorticoid receptor antagonists target fibrosis-related myocardial stiffening, with antifibrotic and endothelial effects that may improve indices of diastolic function (4, 5, 74, 75). The clinical picture differs by agent. The steroidal agent spironolactone produced only modest, largely regionally driven benefit in TOPCAT, limited to reductions in heart failure hospitalization without a clear mortality benefit (74, 75). The non-steroidal agent finerenone, by contrast, significantly reduced the composite of cardiovascular death and total worsening heart failure events in HFmrEF and HFpEF in FINEARTS-HF, providing the first definitive evidence of benefit from this class across this ejection fraction range (76, 112). A recent Bayesian network meta-analysis comparing finerenone, eplerenone, and spironolactone further contextualizes their relative efficacy and safety in HFpEF (77).

The CORMICA trial demonstrated benefit from tailored CMD treatment based on structural and functional dysfunction in patients with INOCA (57). Patients categorized into the vasospastic angina subgroup, defined by an endothelial-dependent CFR below 1.5 on acetylcholine challenge, showed improved symptoms with calcium channel blockers and nitrates (57). Conversely, patients in the structural subgroup with CFR below 2 and IMR above 25 without vasospasm had the greatest symptomatic improvement with beta blockers, particularly nebivolol, currently being investigated in the MOTORS trial (57, 78). This trial was not extrapolated to HFpEF patients and was limited to patients with CMD. While these medications have limited proven benefit in HFpEF generally, they may show promise in established CMD within the HFpEF population, especially in patients with concomitant angina.

### 5.2 Novel pharmaceuticals with potential in HFpEF as they relate to CMD

HFpEF heterogeneity has led to various trials illustrating the potential of nuanced, non-conventional medications in individualized treatment.

Mitochondrial energetics have become one target of HFpEF therapy. HU6 is a controlled metabolic accelerator that increases mitochondrial inefficiency in adipose tissue by enhancing proton leak, studied in the HuMAIN-HFpEF trial (79). While showing modest but significant reductions in body weight and visceral adiposity, no significant improvements in exercise capacity or quality of life metrics were seen in this small trial over 19 weeks (79). Its predominant potential effect on CMD relates to arterial elastance and ventriculoarterial coupling through pericardial fat loss. Nicotinamide riboside has shown, in preclinical models, the capacity to decrease inflammatory cytokines and improve aerobic capacity by reducing mitochondrial damage and reactive oxygen species (80). These data are preclinical and have not yet been confirmed in a human efficacy trial. Myeloperoxidase inhibition evaluated in the SATELLITE trial showed no clinical benefit in cardiovascular function, demonstrating that mechanistic plausibility has not translated into proven utility (81).

Medications influencing the NO-cGMP-PKG pathway, and thereby endothelial dysfunction in CMD, include soluble guanylate cyclase stimulators and activators, phosphodiesterase inhibitors, and inorganic nitrates and nitrites (26, 82–84). Soluble guanylate cyclase activation and phosphodiesterase inhibition can, in principle, restore PKG activity and reduce passive stiffness via titin phosphorylation, potentially improving the diastolic compromise in HFpEF that arises from endothelial-dependent CMD (26, 82–84). Agents acting on this axis have been evaluated in HFpEF, including the phosphodiesterase-5 inhibitor sildenafil in RELAX, the soluble guanylate cyclase stimulator vericiguat in VITALITY-HFpEF, and the soluble guanylate cyclase stimulator praliciguat in CAPACITY-HFpEF (85–87, 114, 115, 118). None has demonstrated significant clinical benefit, so the promise of this pathway remains mechanistic rather than proven.

Pro-angiogenic and vascular remodeling strategies through CD34-mediated cell therapy have shown validity in improving CFR through microvascular regeneration, decreasing microvascular rarefaction (13, 88). The HERMES trial highlights interleukin-6 inhibition with ziltivekimab as a potential mediator of reducing inflammation (82). Similarly, colchicine and urate transporter-1 inhibitors are being studied in CMD and HFpEF, as hyperuricemia and xanthine oxidase mediated oxidative stress can compromise CFR through systemic inflammation and endothelial dysfunction (89, 90). Myosin inhibition with agents such as mavacamten may theoretically improve ventriculoarterial coupling and has shown improvement in biomarkers such as troponin and N-terminal pro B-type natriuretic peptide in HFpEF populations in the EMBARK trial (91).

Trials targeting the endothelin and prostacyclin axes in HFpEF with pulmonary vascular disease have so far been disappointing rather than promising. The endothelin receptor antagonist macitentan did not improve N-terminal pro B-type natriuretic peptide or heart failure outcomes in the SERENADE trial and was associated with more fluid retention and worsening heart failure (92). The oral prostacyclin analog treprostinil program (SOUTHPAW) was terminated early for slow enrollment, which precluded an efficacy analysis. These experiences show how difficult it has been to translate mechanistically attractive vasodilator strategies into clinical benefit in HFpEF, and they argue for phenotype-enriched patient selection in future studies.

### 5.3 Current and future models of surgical intervention in refractory HFpEF

In refractory CMD and HFpEF, new surgical options [Figure 4] may benefit patient morbidity. The COSIRA and COSIRA-II trials use coronary sinus reducer devices in refractory angina patients to redistribute coronary venous outflow and improve subendocardial perfusion (93, 94). While COSIRA showed potential benefit in obstructive coronary artery disease, COSIRA-II is ongoing and shows potential for addressing CMD through sinus narrowing (93, 94). Cardiac contractility modulation, currently being evaluated in the AIM HIGHer trial, has not yet demonstrated statistically significant improvement in all-cause mortality or heart failure hospitalizations (95, 96). However, a European pilot study using non-excitatory electrical impulses to the myocardium did show improvement in Kansas City Cardiomyopathy Questionnaire scores, left atrial volume index, and *E*/*e*′ (95, 96).

Atrial shunting devices rest on the pathophysiologic basis that pulmonary hemodynamics improve from shunting through decreased pulmonary capillary wedge pressure with decreased augmentation of pulmonary artery systolic pressures, increased oxygen delivery to pulmonary vasculature, and increased flow leading to better recruitment (97). Trials have mixed results, with RELIEVE-HF showing worse outcomes in the HFpEF population and REDUCE LAP identifying a group of super-responders who also had no pulmonary vascular remodeling (97–99). The primary discrepancy appears to relate to N-terminal pro B-type natriuretic peptide values, with the RELIEVE-HF population averaging approximately 1800 and the REDUCE LAP population approximately 300 (98, 99). This may reflect the extent of HFpEF progression or phenotype in the populations studied. The RESPONDER-HF trial seeks to study the super-responder group and clarify the utility of atrial shunting devices (100).

Pericardial resection devices use thoracotomy or a subxiphoid approach to mechanically reduce the pericardial fat pad or remove pericardial constraint, increasing diastolic filling reserve and reducing external restraint on the heart (101). Left ventricular expanders achieve similar physiologic goals by mechanically enlarging the left ventricle in HFpEF patients with severe concentric remodeling (102). These devices remain in early feasibility trials.

### 5.4 Inotropic and vasopressor considerations in septic shock for CMD-HFpEF populations

Evidence regarding the effects of vasopressors on the cardiovascular system in patients with both HFpEF and CMD during septic shock remains limited. Because of a diminished capacity of the coronary circulation to augment myocardial blood flow and prevent ischemia, achieving target mean arterial pressure and cardiac index in septic shock does not guarantee optimal myocardial perfusion in this population (15, 103). Additionally, medications that increase afterload may precipitate acute subendocardial ischemia because of baseline impairment in cardiac-coronary coupling (103). Impaired myocardial relaxation in HFpEF further exacerbates this risk, as vasopressors reduce diastolic relaxation, intensifying diastolic dysfunction and restricting early diastolic coronary perfusion, when myocardial blood flow peaks during the cardiac cycle (103). This sequence may establish a detrimental cycle in which increased afterload from vasopressors aggravates diastolic dysfunction, further diminishing subendocardial blood flow.

Norepinephrine is considered the first-line vasopressor in septic shock. Its mechanism involves alpha-1 adrenergic-mediated vasoconstriction and activation of the beta-adrenergic pathway. Increasing peripheral vascular resistance through alpha-1 receptors may exacerbate CMD. As peripheral vascular resistance rises, coronary microvascular resistance may also increase, potentially reducing coronary flow reserve, although this relationship remains controversial (4, 103, 104). Norepinephrine also facilitates beta-2 receptor mediated coronary dilation, which may counterbalance acute changes in coronary flow reserve (103, 104). Cardiac sympathetic imaging with 123I-meta-iodobenzylguanidine, which mimics norepinephrine pharmacokinetics, was studied in women with CMD (105). That study identified a low late heart-to-mediastinum ratio below 1.6, which suggests potential increased risk of ventricular arrhythmias in this subgroup (105).

Evidence for inotropic support with dopamine and dobutamine is mixed. Although clinical evidence is lacking, these agents may theoretically induce a coronary supply–demand mismatch and ventriculoarterial uncoupling by directly increasing myocardial oxygen consumption through enhanced contractility and heart rate, thereby decreasing subendocardial oxygenation (15, 106). This increased cardiac workload may predispose patients with HFpEF to acute subendocardial ischemia. Milrinone has positive lusitropic effects and, in a single-center study, showed a significant reduction in pulmonary capillary wedge pressure after a 20-min infusion in stable HFpEF patients (107, 108). The vasoplegia potentiated by milrinone, however, complicates its utility in sepsis.

Vasopressin may offer theoretical benefits as it acts through V1 receptors to increase peripheral vascular resistance with minimal impact on pulmonary vascular resistance (104, 106). This mechanism may preserve subendocardial perfusion as reduced right ventricular afterload could preserve ventriculoarterial coupling. However, evidence regarding direct effects on cardiac vasculature is limited and at times contradictory.

Levosimendan, a calcium sensitizer and ATP-sensitive potassium channel opener with inotropic and vasodilatory effects, does not significantly increase intracellular calcium or myocardial oxygen demand and is not currently available in the United States. It has shown utility in improving pulmonary pressures, right ventricular function, and unstressed blood volume, as well as improved coronary blood flow and reduced filling pressures (109–111). The HELP-PH-HFpEF trial demonstrated a 30-m improvement in 6-min walk test distance with levosimendan compared with placebo in HFpEF patients (111). Conceptually, it could improve microvascular perfusion in CMD-HFpEF, but its vasodilatory component, such as that of milrinone, creates uncertainty in sepsis, with no clinical trials or guidelines established for this application. It could have potential depending on dose-specific pharmacodynamics, with a dose of 0.1 micrograms per kilogram per minute showing possible utility (109, 110).

Recognizing that this remains conceptual, we suggest that earlier addition of vasopressin as an adjunct to wean norepinephrine may be reasonable in CMD-HFpEF patients with low-grade septic shock. The addition of milrinone or levosimendan may be useful in mixed cardiogenic and septic shock states. This approach is non-specific and requires significant clinical judgment based on individual patient hemodynamics. Advanced hemodynamic assessment, whether invasive or non-invasive, using metrics such as cardiac index, pulmonary capillary wedge pressure, central venous pressure, dP/dt, and dynamic arterial elastance, can guide vasopressor and inotropic management, with more research needed to assess clinical utility and the short- and long-term effects of different combinations on CMD.

## 6 Conclusion

The epidemiological and pathophysiologic association between CMD, both structural and functional, and HFpEF has inherent potential for identifying new pharmaceutical and surgical interventions to reduce patient morbidity, heart failure hospitalization rates, and potentially major adverse cardiovascular events, although no proven causal correlation has been established. Early diagnosis through invasive functional angiography or non-invasive means, alongside HFpEF assessment, offers potential for guiding individualized treatment plans based on phenotypic cluster probability analysis. A combination of established medications such as sodium-glucose cotransporter-2 inhibitors with emerging treatments including interleukin-6 inhibitors and left ventricular expanders, with various ongoing trials, will define the future of precision medicine in targeting CMD in HFpEF.

Sepsis shares many mechanistic links to CMD and creates a pathophysiologic state that we hypothesize can potentiate a second hit, with downstream effects during and after hospitalization. However, sepsis within CMD-HFpEF populations remains poorly understood, and the associations described here are hypothesis-generating rather than established.

Sepsis-associated CMD appears reversible, improving or resolving on limited imaging follow-up. This contrasts with the fixed, remodeling-based microvascular injury of chronic HFpEF, and suggests that the two operate at least in part through distinct and temporally separable mechanisms. If so, they may be addressable through complementary strategies given simultaneously: one directed at the acute, reversible, predominantly functional dysfunction of sepsis, for example, vasomotor tone and NO and oxidative stress pathways, and one directed at the chronic, structural CMD of HFpEF, including anti-inflammatory, antifibrotic, and metabolic-remodeling targets. This dual, mechanism-specific framing further strengthens the case for a precision-medicine approach in the septic HFpEF patient.

Analyses using comprehensive hemodynamic assessment and orthogonal polarization spectral imaging can be applied in future studies to more clearly identify the effects of sepsis on CMD, especially in shock states. Prospective, phenotype-aware evaluation, including attention to sex-specific differences, represents the clearest path forward.

## Glossary

ACE/ARB: Angiotensin-converting enzyme inhibitor/angiotensin II receptor blocker
Ach: Acetylcholine
ANOCA: Angina with non-obstructive coronary arteries
ARNI: Angiotensin receptor neprilysin inhibitor
BNP/NT-proBNP: B-type natriuretic peptide/N-terminal pro B-type natriuretic peptide
CAD: Coronary artery disease
CFR/eCFR: Coronary flow reserve/endothelial-dependent coronary flow reserve
CI: Cardiac index
CMD: Coronary microvascular dysfunction
CO: Cardiac output
CPET (with RHC): Cardiopulmonary exercise testing (with right heart catheterization)
CRP: C-reactive protein
CVP: Central venous pressure
cGMP: Cyclic guanosine monophosphate
Ea/Ees: Arterial elastance/ventricular end-systolic elastance
EDHF/EDH: Endothelium-derived hyperpolarizing factor/endothelium-dependent hyperpolarization
eNOS/iNOS/nNOS: Endothelial/inducible/neuronal nitric oxide synthase
ET-1: Endothelin-1
GLP-1 RA: Glucagon-like peptide-1 receptor agonist
GLS: Global longitudinal strain
HFpEF/HFmrEF/HFrEF: Heart failure with preserved/mildly reduced/reduced ejection fraction
HMR: Heart-to-mediastinum ratio (cardiac MIBG imaging)
ICU: Intensive care unit
IMR: Index of microvascular resistance
INOCA: Ischemia with non-obstructive coronary arteries
KCCQ: Kansas City Cardiomyopathy Questionnaire
LGE: Late gadolinium enhancement
LV/RV/LA: Left ventricle/right ventricle/left atrium
MACE: Major adverse cardiovascular event
MBF/MFR: Myocardial blood flow/myocardial flow reserve
MIBG: 123I-meta-iodobenzylguanidine
MINOCA: Myocardial infarction with non-obstructive coronary arteries
MPO: Myeloperoxidase
MRA: Mineralocorticoid receptor antagonist
NO: Nitric oxide
OCTA: Optical coherence tomography angiography
OPS: Orthogonal polarization spectral (imaging)
PCWP: Pulmonary capillary wedge pressure
PDE: Phosphodiesterase
PET: Positron emission tomography
PKG: Protein kinase G
PVD: Pulmonary vascular disease
RAAS: Renin–angiotensin–aldosterone system
ROS: Reactive oxygen species
SGLT2i: Sodium-glucose cotransporter-2 inhibitor
sGC: Soluble guanylate cyclase
SvO2: Venous oxygen saturation
TDI: Tissue Doppler imaging
TGF-beta: Transforming growth factor beta
TNF-alpha/TNFR1: Tumor necrosis factor alpha/TNF receptor 1
VAC: Ventriculoarterial coupling
VO2: Oxygen consumption
ZSF1: Zucker fatty/spontaneously hypertensive rat (model of cardiometabolic HFpEF)
